# National programme for prevention of burn injuries

**DOI:** 10.4103/0970-0358.70716

**Published:** 2010-09

**Authors:** J. L. Gupta, L. K. Makhija, S. P. Bajaj

**Affiliations:** Department of Burns, Plastic & Maxillofacial Surgery, Safdarjung Hospital, New Delhi; 1 PGIMER & Dr R M L Hospital, New Delhi, India

**Keywords:** National programme for prevention of burn injuries, organized burn care

## Abstract

The estimated annual burn incidence in India is approximately 6-7 million per year. The high incidence is attributed to illiteracy, poverty and low level safety consciousness in the population. The situation becomes further grim due to the absence of organized burn care at primary and secondary health care level. But the silver lining is that 90% of burn injuries are preventable. An initiative at national level is need of the hour to reduce incidence so as to galvanize the available resources for more effective and standardized treatment delivery. The National Programme for Prevention of Burn Injuries is the endeavor in this line. The goal of National programme for prevention of burn injuries (NPPBI) would be to ensure prevention and capacity building of infrastructure and manpower at all levels of health care delivery system in order to reduce incidence, provide timely and adequate treatment to burn patients to reduce mortality, complications and provide effective rehabilitation to the survivors. Another objective of the programme will be to establish a central burn registry. The programme will be launched in the current Five Year Plan in Medical colleges and their adjoining district hospitals in few states. Subsequently, in the next five year plan it will be rolled out in all the medical colleges and districts hospitals of the country so that burn care is provided as close to the site of accident as possible and patients need not to travel to big cities for burn care. The programme would essentially have three components i.e. Preventive programme, Burn injury management programme and Burn injury rehabilitation programme.

## INTRODUCTION

Organized burn care is possible in developed world because there is direct or indirect involvement of the government, universal health insurance cover, legislation like right to health is in place and high level of safety awareness in public. Such luxuries do not exist in developing countries like India. India, the second most populous country in the world with over a billion people has an estimated annual burn incidence of 6-7 million, based on data from major hospitals when extrapolated to whole of the country, which is the second largest group of injuries after road accidents. Nearly 10% of these are life threatening and require hospitalization. Approximately 50% of those hospitalized succumb to their injuries. Nearly 1 to 1.5 lac people get crippled and require multiple surgeries and prolonged rehabilitation. Seventy percent of the burn victims are in most productive age group of 15 to 40 years and most of the patients belong to poor socioeconomic strata. All these figures are approximate figures as we have no national data on burns as it is not a notifiable disease and central registry is nonexistent. The burn scenario is grave not only due to the high incidence but is also compounded by absence of organized burn care at primary and secondary health care level. But there is a silver lining to this grim situation, that 90% of all burn injuries are preventable. However, absence of stringent law regarding environmental safety, substandard manufacturing of household electric and cooking equipments and general lack of safety consciousness fail to curb this preventable menace. The economic aspect of burns also needs to be addressed. The prohibitive cost of treatment and rehabilitation and most victims being poor explain the gross shortage of specialized burn units in private sector and reluctance on the part of the private players to share the burden. Even in the capital city of Delhi, there are less than 200 beds for burn management to cater to the need of a population of over 14 million. The recent rise in the incidents of terrorist activities and manmade disasters, contributing to quantum jump in Burn Injury cases also calls for national preparedness to cope with the challenge of this Public Health Problem.

## ROLE OF NATIONAL ACADEMY OF BURNS INDIA

Over the years no government realized the gravity of the situation so not many burn centers were started in the last 40-50 years. In 1992, like minded burn care professionals formed a professional association, National Academy of Burns-India. Its aim was to reduce the incidence of burns by generating safety awareness amongst masses and to improve the burn care through research and training of surgeons, nurses and paramedical staff. Under the aegis of this organization, academic activities like conferences, CMEs, workshops, training, a national journal and public awareness programme through radio and TV were started. These efforts reaped some benefit as many more surgeons got involved in burn management, but still organized burn care failed to reach remote areas and remained confined to major cities. Realizing that the situation can not be improved without the involvement of the government, representations on behalf of NABI were made to the Prime Minister and Ministry of Health and Family Welfare about the situation of burn care in the country. It was impressed upon that considering the magnitude of the problem of burn injuries, the deficiency in our public healthcare delivery system and absence of prevention programme there is an urgent need of setting up a national programme with a multi pronged preventive strategy to reduce incidence, formulate cost effective treatment and rehabilitation protocol and set up a central burn registry.

Considering the request Director General of Health Services constituted a core group under the chairmanship of Addl. DG to prepare and submit a proposal for National Programme for Burn injuries. After a number of meetings and deliberations a proposal for National Programme for Prevention of Burn Injuries (NPPBI) was prepared and submitted. The Secretary Health has approved the proposal and agreed in principle to start the Programme. The programme would be implemented in a phased manner in whole of the country.

## THE GUIDING PRINCIPLES OF THE NPPBI

The National Programme for Burn Injuries shall be set up and function with the following Guiding Principles:

- To optimally and effectively utilize the existing health care and allied sectors services and infrastructure
- To elicit participation of Government, Non Government and private/corporate sector to avoid duplication of efforts and expenditures
- To evolve three tier programme structure to have universal coverage and reach
- The Programme shall have balanced focus on preventive, curative and rehabilitative aspects of Burn Injuries
- To establish a central burn registry

## PROGRAMME STRUCTURE OF NPPBI

To establish and manage the programme, a defined programme management will be structured under the following headings:

- Programme Division in Directorate General of Health Services
  A programme officer with Public Health / Health
  Promotion and Education qualifications
  Consultants in Burns, Rehabilitation and Health promotion and Education specialties
  Requisite support staff.
- A National Burn case reporting mechanism
  Utilizing MIS in the country from Sub centre> PHC>CHC >District Admn.>State> Centre.
- A co-ordination committee

Experts from Burns and Plastic surgery, Physical medicine and Rehabilitation, Health education and Health promotion to guide, oversee, supervise and evaluate the NPPBI programme activities.

## PARTNERS IN THE PROGRAMME

In the present set-up there are many players working directly or indirectly for prevention of Burns and other traumas, First aid and transportation facilities. Their services will be continued to be utilized for further improvements in the burn care. Various departments involved are:

- MOH and FW through its Health services infrastructure e.g. Departments of Burns and plastic, causality services (Secondary and tertiary prevention/curative care)
- Fire Services, Ministry of Home Affairs
- Education Department (HRD)
- Women and Child Ministry (ICDS)
- Local Self Governments (PRI)
- Civil Defence and Home Guards (Ministry of Home Affairs)
- Police Department
- Others

## THE PROGRAMME STRATEGY OF NPPBI

To address the various dimensions of the problem of Burn Injuries and to fill the identified gaps in the existing public health care delivery system the “National Programme for Prevention of Burn Injuries” requires multi pronged strategy. The programme would essentially have three components:

1. Preventive programme
2. Burn injury management programme
3. Burn injury rehabilitation programme

### Preventive programme

The key strategies of this programme would be:

- Development and implementation of an appropriate, need based communication/ bcc strategy.
- Advocacy with the Policy makers, Administrators in Health and Allied Sectors, Corporate and Industry sectors.
- Developing key messages for general public and special target groups such as healthcare providers at primary, secondary and tertiary level for vulnerable groups e.g women and children, industrial workers and so on. It would be necessary to maintain consistency in the messages through out.
- Media engagement strategy
- A National commemorative day/ week for prevention of Burn Injuries
- Communication organizational structure at centre, state, district, PHC level
- Capacity building programme for IEC/ BCC manpower at all the three levels
- Monitoring and Evaluation of Communication interventions for prevention

#### Programme activities

- Audience research (Formative research): To be undertaken by institution like ICMR/MCI to provide a base line data pertaining to the specific behavior to be changed for communication/BCC strategy.
- Advocacy with policy makers, administrators, M.P/MLA/Local Self Govt.
- Campaigns during parliament/Assembly sessions. Printing informative brochures etc.
- Networking with active partners: Bureaucrats, Stakeholders from Private and Corporate sectors and NGOs, Professional associations.
- Integration with ongoing educational and training activities
- Fulfilling personnel requirement at different levels
- Capacity development of available manpower
- Health communication through media mix as per local needs
- Commemorative Burn Prevention week to be observed across the country

Monitoring and evaluation: Routine monitoring shall be done through existing monitoring system at National and State level or evaluation by an external agency

### Burn injury management programme

The main strategies for Burn care would be to provide physical infrastructure and manpower for Burn care at all three levels of health delivery system through inter agency coordination and Public private partnership. This will be achieved through provision of material resources and capacity building programmes for easy accessibility, equity and universal coverage. Quality management and Monitoring would be done to ensure maintenance of standards.

For quality burn injury management at all the three levels of healthcare delivery system there would be certain additional requirement of following:

#### Primary level

The existing space available will be utilized for various activities and the existing staff (such as ANMs, nurses, dressers, etc.) will be sensitized and trained on burn first aid.

#### District level

A small 4-6 bedded Burn unit will be set up in district hospitals. The existing operation theatre and other facilities will be utilized but will be augmented by providing basic equipment such as Vital parameter monitor, Skin graft mesher, Humby’s knife, Portable light etc. and partly the consumables will also be provided. One Advanced Life Support Ambulance per district with manpower will be provided.

#### Tertiary level

Capacity building will be done where there are no existing facilities or strengthening will be done if there are some existing facilities by providing additional infrastructure and manpower. A Burn Unit of 12 beds including an ICU of 4 beds will be created. The existing operation theatre and other facilities will be utilized but will be augmented by providing basic equipment such as Ventilators, Vital parameter monitors, Skin graft mesher, Humby’s knife, Portable lights, Dermatome etc.

#### Training programme for capacity building of burn care manpower

For smooth and efficient Burn care management capacity building of the relevant healthcare manpower would be undertaken as per plan detailed below:

- The Surgeons/Medical officers at the district level, two from each district shall be trained by the Burns and Plastic surgeons at the medical college/ Selected Training Centers.
- Training of the district level workers will be done at district hospitals by the trained Surgeons and Medical officers in Burn care.
- Orientation training of the primary level workers will be done at district centre by the Surgeon /Medical Officers.
- On the job Training of the medical college workers will be done at existing burn centers routinely.

### Burn injury rehabilitation programme

The essential components of Rehabilitation of Burn Injuries would encompass development of infrastructures- physical, manpower and materials with capacity and skill development initiatives. It will be equitable, accessible and with universal outreach. Inter agency coordination and public private partnership will be used. Monitoring and quality control will be done. Services to be provided at three levels will be as follows:

#### Primary level

The main stress would be to integrate with the villagers through the Aanganwadi workers and other community level functionaries for awareness generation regarding scar management, therapeutic exercises with home exercise programme, splinting, electrotherapy modalities (Cold, Heat etc.) and activities of daily living (Modifications and equipments).

#### District level

To set up fully equipped Burns Rehabilitation Centre in approximately 300sq ft area with a basic team consisting of a surgeon, 3 Physiotherapist/occupational therapist and members like multi rehabilitation workers who can be a pivotal link between the three tiers of the system. The services of a psychologist and vocational counselor can be utilized on fixed days basis. Equipment such as diathermy, ultrasound, electrical stimulators and traction equipments will be provided.

#### Tertiary level

At this level every possible facility and information related to complete burn rehabilitation should be available. This includes issues on pre and post operative assessments and planning, execution of treatments. Disability certificates, vocational guidance and placements, monetary assistance through various resources, functional prosthesis and compilation of various Government and Non Government organizations working in this sector. A special group of Burn care professionals should be allotted with the work of patient out come assessments.

## SETTING UP OF NATIONAL REGISTRY FOR BURNS

A National Burn case registry mechanism will be set up utilizing MIS in the country from sub centre> PHC>CHC >District Admn.>State>Centre. This will be very useful data in future planning of various strategies for prevention, burn management, behavioral or tradition/cultural changes required, safety regulations etc. to reduce the burn incidence and improve burn care in the country.

## IMPLEMENTATION OF THE PROGRAMME

To begin with the programme will be initiated in few states with few Medical colleges and their adjoining Districts. Then in the next five year plan it will be rolled out in all the medical colleges and districts hospitals of the country so that burn care is provided as close to the site of accident as possible and patients need not to travel to big cities for burn care.

## CONCLUSION

In a developing country like India with a population of more than 1.2 billion and a very high incidence of burn injuries, lack of life insurance cover and megre facilities for burn care in government as well as private sectors, a national initiative in the form of National Programme is required to provide organised burn care at village level. When National Programme for Prevention of Burn Injuries is in place there are going to be preventive activities at the lowest level of health delivery system and even first aid facilities will be available at this level. We will have burn care management and rehabilitation facilities by trained manpower in each and every district hospital and Medical College of the country. There will be about 700 to 800 dedicated ICU beds and total 6000 beds available for burn care. Advanced ambulance for transportation of patient from accident site to appropriate burn unit will be available in the remotest areas. Besides this we will also be able to help the government in formulating policies for environmental safety for industrial workers, manufacturing safety and quality regulation of cooking and other appliances etc. We will also have a national burn registry to collect and compile the data for analyzing the causative factors, behavioral and social patterns and any other factors responsible for high incidence of burn injuries and will also help us in planning future strategies for prevention and management of burn injuries. We hope that with National Programme for Prevention of Burn Injuries we will be able to reduce the incidence and improve the care for burn injuries in the country.

